# Fermented whey-based product improves the quality of life of males with moderate lower urinary tract symptoms: A randomized double-blind study

**DOI:** 10.1371/journal.pone.0191640

**Published:** 2018-02-23

**Authors:** Kristo Ausmees, Kersti Ehrlich-Peets, Mirjam Vallas, Andre Veskioja, Kadi Rammul, Aune Rehema, Mihkel Zilmer, Epp Songisepp, Tiiu Kullisaar

**Affiliations:** 1 Department of Urology, Medita clinic, Tartu, Estonia; 2 BioCC LLC, Tartu, Estonia; 3 Institute of Biomedicine and Translational Medicine, University of Tartu, Tartu, Estonia; Texas Technical University Health Sciences Center, UNITED STATES

## Abstract

**Purpose:**

The purpose of this research was to evaluate the effect of a specific fermented whey product on lower urinary tract symptoms, main prostate related indices and oxidative stress/inflammatory markers in urine and seminal plasma in men with moderate dysuric symptoms. An additional purpose was to clarify associations between different parameters with special emphasis on pain.

**Methods:**

This was a prospective randomized double-blind 4-weeks study on men with moderate lower urinary tract symptoms who underwent the evaluation for quality of life at the baseline and at the end of the study. The symptoms were characterized by International Prostate Symptom Score (I-PSS) and National Institutes of Health Chronic Prostatitis Symptom Index (NIH-PSI), the maximum urinary flow and the main prostate-related indices. In order to obtain more comprehensive information about the effects of fermented whey product on systemic oxidative stress marker 8-EPI and seminal plasma inflammatory markers (interleukin-6 and interleukin-8) were also measured.

**Results:**

After 4 weeks consumption of fermented whey product there was a statistically significant decrease of prostate-specific antigen level in serum and systemic stress marker 8-EPI in urine compared to control group. Maximum urinary flow and NIH-PSI all studied scores and sub-scores had also significant improvement. In addition, seminal plasma interleukin-8 level substantially decreased.

**Conclusions:**

The consumption of special fermented whey product improved urinary function, reduced lower urinary tract symptoms, systemic oxidative stress marker and seminal plasma inflammatory status. Thus it contributed to an improvement of the quality of life in men with moderate lower urinary tract symptoms.

## Introduction

Benign prostatic hyperplasia (BPH) is often associated with lower urinary tract symptoms (LUTS) characterized by irritative, obstructive and nocturnal symptoms. It is well accepted that men with LUTS are classified as mild, moderate and severe symptoms. A large number of men have moderate LUTS which impairs their general quality of life (QoL).[1] It has been shown that LUTS is related to increased inflammation and oxidative stress (OxS).[2] Therefore, there is a need for new safe, well-tolerated and clinically accepted nonpharmacological products for men with LUTS.

It is shown that hydrolyzed whey protein isolate is able to increase the level of central intracellular antioxidant glutathione and protect from oxidative damage.[3] The whey proteins are biologically valuable because of their high content of essential branched-chain amino acids (BCAAs) and a sulfur-containing amino acid cysteine, the rate-limiting precursor of glutathione synthesis.[4–5]

The main aim of the present study was to evaluate the effect of a special fermented whey-based product (FWP) on men with moderate LUTS. For this purpose data of International Prostate Symptom Score (I-PSS) and National Institutes of Health Chronic Prostatitis Symptom Index (NIH-CPSI) scores with special emphasis on pain sub-score, main prostate related characteristics, including urinary function and prostate-specific antigen (PSA) level were collected. Systemic OxS and seminal plasma inflammatory status were also measured to evaluate biological effects of FWP.

## Material and methods

### Study population

**Fig 1:**
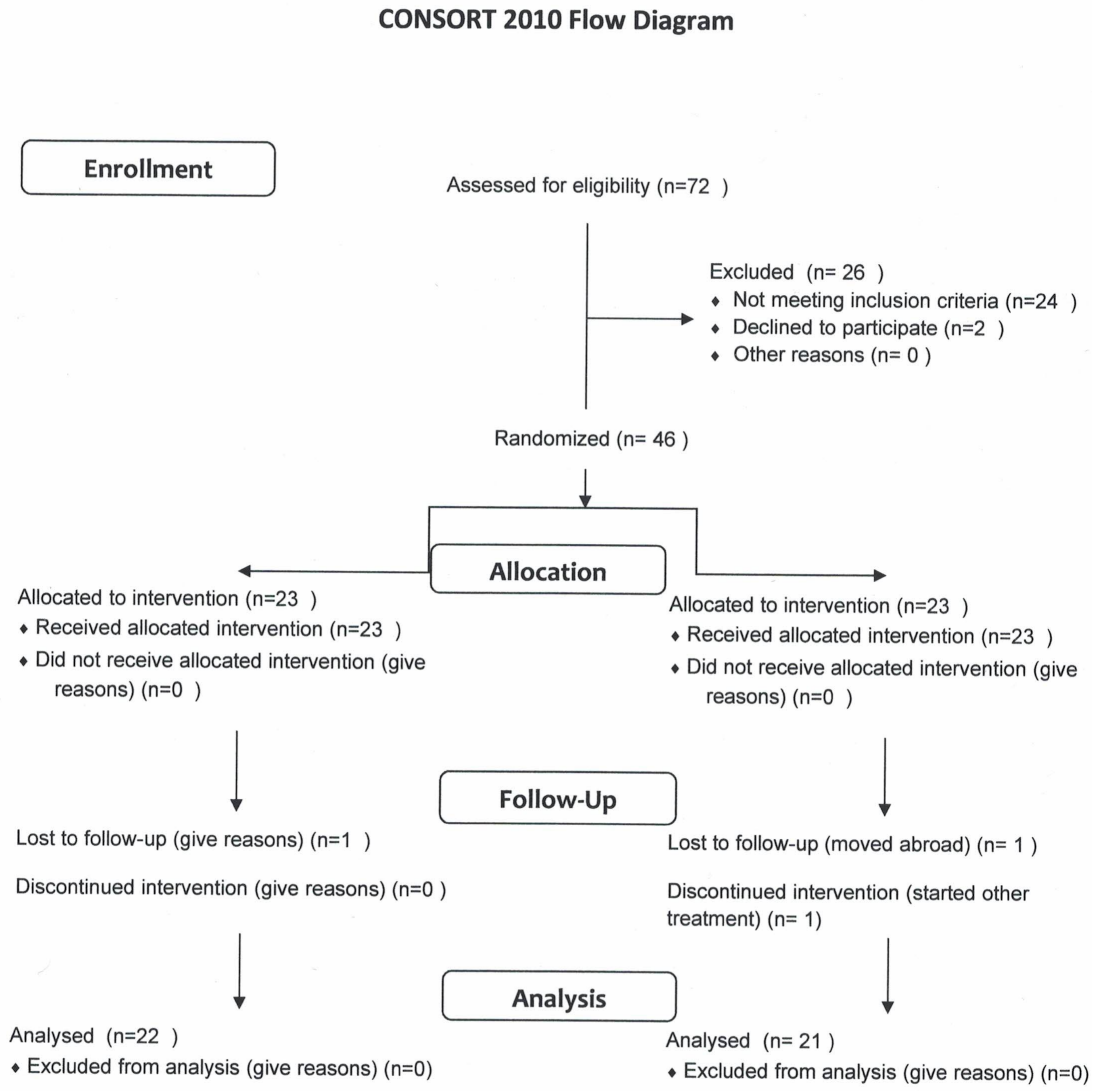
The CONSORT-type scheme of the handling of subjects.

In the initial phase (between September 2012 and November 2014), 46 men with moderate urinary tract symptoms (I-PSS score 8–19), who underwent the screening for prostate health at the urology department of Medita clinic (Teguri 37b, Tartu, Estonia, www.medita.ee), were recruited into a randomized double-blind study (Fig 1).

The inclusion criteria for the study were total serum PSA level of ≤ 10 ng/mL, maximum urinary flow (Q_max_) of 5–15 mL/s, minimum voided volume ≥ 125 mL and maximum post-voided residual urine ≤ 300 mL. Exclusion criteria for this study included history of alcohol or drug abuse, current finding and/or treatment of colic pain, prior or current problems and/or treatment of urogenital tumors, surgery of prostate, chemo- or radiation therapy in the pelvic region, abnormal findings from a digital rectal examination and common urinary pathogens and/or white blood cells count >1 million/mL in seminal plasma and/or urine at the screening-phase of study. In addition, none of the study subjects experienced fever accompanying pelvic pain symptoms, acute urinary retention nor received therapy with α1-blockers or 5α-reductase inhibitors muscarinic receptor antagonists and/or phytotherapy within three months prior the study. All subjects were of age ≥ 30 years. Among these subjects, one man taking α1-blockers within the study period was excluded. Also, 2 subjects withdrew from the study.

Finally, the conclusive number of subjects was 46. They were randomized into two groups, the study-group (n = 23) consumed 200 g/day of fermented liquid whey product and 23 men in the control-group consumed control product 200 g/day during 4 weeks. 22 men in the study group and 21 men in the control group concluded the protocol. Randomization (4-member blocks, letters A and B were used) was performed by a person who was not familiar with clinical data—and other investigators were not aware of the product the subjects got, A or B. The products were in otherwise identical bottles, just marked A or B. Until the basic statistics was performed, the nature of A and B (FWP or control drink) was not revealed to any of the investigators, the only persons knowing the information where the ones included in production of the products.

### Isoprostanes (8-EPI) in urine

A first morning mid-stream urine sample was obtained between 8 a.m. and 10 a.m. The samples were centrifuged and stored until analysis at -80°C. The method of determination of 8-isoprostanes (8-EPI) in biological samples has been described and accepted (BIOXYTECH 8-Isoprostane Assay, Cat. No.21019; Oxis International, Inc., Portland, OR, USA).^3^

### Prostate-related characteristics

At the baseline of the study and after 4-week treatment, all participants completed the questionnaires, including the NIH-CPSI and I-PSS for LUTS.[6–7] All subjects were assessed for total prostate volume (TPV) by trans-rectal ultrasonography (using Logiq 5 Pro by GENERAL ELECTRIC, Milwaukee, WI, USA) and for urinary flow rates by uroflowmetry (using Urodyn 1000 by MEDTRONIC, Minneapolis, MN, USA).

### Seminal plasma analysis

Routine seminal plasma analysis was performed according to WHO guidelines to detect inflammatory markers interleukin-6 (IL-6) and interleukin-8 (IL-8).[8] Semen was obtained by masturbation and ejaculated into a sterile collection tube in a private room near the laboratory. The recommended abstinence period was a minimum of 48 hours but not longer than 7 days.

IL-6 and IL-8 levels in seminal plasma (100 μl of specimen was required for the assay) were measured at the Synlab Laboratories Inc (Tartu, Estonia, www.synlab.ee), using the Immulite automated chemiluminescence immunoassay analyzer (Immulite Siemens Healthcare Diagnostics Inc, Deerfield, IL, USA) according to the manufacturer’s instructions.

### Blood samples

Venous blood was obtained from the cubital vein between 8 a.m. and 11 a.m. after overnight fasting or a light morning meal. The samples were centrifuged, serum was isolated and PSA was detected within 2 hours at the Synlab Laboratories Inc. The level of PSA in serum was measured using the Immulite automated chemiluminescence immunoassay analyzer (Immulite Siemens Healthcare Diagnostics Inc, Deerfield, IL, USA) according to the manufacturer’s instructions.

### Fermented whey-based drink production

Pasteurized and chilled whey from semi-hard cheese production (cheese dust and fat removed, pH 6.4–6.6) was heated to fermentation temperature (37°C±0.5°C) and patented *Lactobacillus*. *plantarum* MCC1 (DSM 23881) and *L*. *gasseri* MCC2 (DSM 23882), strains with an ability to hydrolyze milk proteins, were added.[9–10] Preparation technology of the FWP is described previously.[11] The parameters of the used whey and fermented end-product are presented in S1 Table.

Water and 12% w/w orange-peach syrup were mixed for producing the control liquid, heated, chilled and bottled the same way as the whey product. The taste of the product for both groups was similar.

### Statistical evaluation

For statistical analyses, Excel (Microsoft, Redmond, WA, USA), and SAS System version 9.2 (SAS Institute, Inc., Cary, NC, USA) software were used. The changes in prostate-related characteristics, seminal inflammatory and urinary OxS markers during the trial were calculated by subtracting the initial measurement value from the measurement value after intervention period. These changes were used for testing differences within and between groups (Wilcoxon signed rank test and Mann-Whitney rank sum test, respectively). Also baseline difference between groups was tested using Mann-Whitney rank sum test. Associations between these characteristics were studied using Spearman product-moment correlation and regression analyses. Multiple regression analyses, including only study group, were performed for changes between baseline and final values and for baseline values. In both regression models LUTS scores were dependent characteristics. Age, main prostate related characteristics (TPV, Qmax, PSA) and urinary OxS marker 8-EPI were independent characteristics in these models.

Multiple regression, including both study and control group, on changes of LUTS characteristics with changes of independent characteristics described above was extended to the model with pairwise interactions of independent characteristics with group.

**Fig 2:**
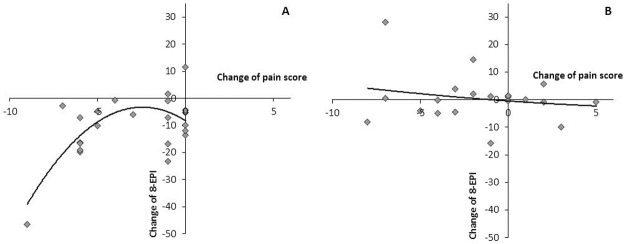
Quadratic relationship between the changes of the pain score and 8-EPI values (R^2^ = 0.446; p = 0.004) in study group after consumption of the fermented whey product.

**Fig 3:**
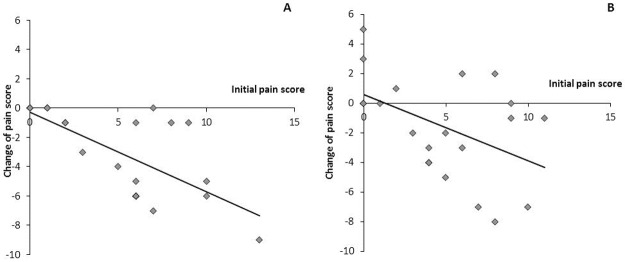
Linear relationship between the initial pain score and the change of the pain score (R^2^ = 0.467; p < 0.001) in study group consuming fermented whey product.

The quadratic regression analysis was performed to evaluate associations between changes of 8-EPI and NIH-CPSI pain sub-score (Fig 2) and linear regression analysis was used to estimate relationship between initial measurement value and change of NIH-CPSI pain sub-scores (Fig 3). Statistical significance was assumed at *p* < 0.05 for all tested parameters.

### Ethical consideration

Participation in the study was voluntary. A written informed consent was obtained from all study subjects. The study was approved by the Ethics Review Committee on Human Research of the University of Tartu (215/T-3) on 29^th^ of May 2012. The trial registration number is ISRCTN51441547. The study conforms to the provisions of the Declaration of Helsinki. The trial was registered retrospectively because of communication issues, but the enrollment of participants did not begin before the positive decision from the ethics committee. The authors confirm that all ongoing and related trials for this intervention are registered.

## Results

The age, different I-PSS and NIH-CPSI sub-scores and inflammatory markers in seminal plasma and OxS markers in the urine of the enrolled subjects are presented in Table 1. At the baseline, there was no statistically significant difference between groups. At the baseline, age as a risk factor for prostate pathologies showed similar correlation with PSA, TPV and IL-8 in seminal plasma as described recently.[12–13] Also, associations between seminal inflammatory markers and prostate-related parameters were comparable with findings of recent studies.[12, 14]

**Table 1:** Prostate related parameters, seminal inflammatory and urinary OxS markers before and after intervention (fermented whey product or placebo).

Characteristics†	Total(n = 43)	Study group(n = 22)		Control group(n = 21)	
	Baseline	Baseline	4 weeks	Baseline	4 weeks
	Mean ± SD	Mean ± SD	Mean ± SD	Mean ± SD	Mean ± SD
Age (years)	54.9 ± 10.2	54.9 ± 11.4		54.8 ± 9.0	
**Basic prostate-related parameters**					
TPV (mL)	35.5 ± 15.8	37.7 ± 19.9	35.0 ± 16.4	33.2 ± 9.8	30.8 ± 12.6
Qmax (mL/s)	11.9 ± 5.4	11.7 ± 2.9	14.2 ± 5.4^1^	12.1 ± 7.2	13.5 ± 6.0
PSA (ng/mL)	3.1 ± 3.4	3.9 ± 3.9	2.6 ± 2.6^2^^,^^3^	2.3 ± 2.7	1.7 ± 1.9^3^
I-PSS subscore (irritative)	5.1 ± 2.5	5.4 ± 2.6	3.2 ± 2.0^2^	4.8 ± 2.5	3.2 ± 2.5^1^
I-PSS subscore (obstructive)	5.6 ± 2.9	6.1 ± 2.7	3.2 ± 2.2^2^^,^^3^	5.0 ± 3.1	3.7 ± 2.9^1^^,^^3^
I-PSS subscore (nocturial)	1.7 ± 1.1	1.9 ± 1.1	1.2 ± 1.1^2^	1.4 ± 1.1	1.3 ± 1.1
I-PSS score (total)	12.0 ± 4.6	13.0 ± 4.5	7.6 ± 4.1^2^	11.1 ± 4.6	8.2 ± 5.3^1^
NIH-CPSI subscore (pain)	5.0 ± 3.6	5.2 ± 3.7	2.1 ± 2.7^2^	4.9 ± 3.5	3.2 ± 3.6
NIH-CPSI subscore (QoL)	2.8 ± 1.2	2.8 ± 1.2	1.9 ± 1.2^2^	2.9 ± 1.3	2.2 ± 1.5
NIH-CPSI score (total)	13.7 ± 6.3	14.0 ± 6.2	7.7 ± 5.1^2^	13.4 ± 6.6	9.6 ± 7.5^1^
**Seminal inflammatory and urinary OxS markers**					
8-EPI in urine (ng/mmol creatinine)	71.5 ± 20.3	68.5 ± 22.1	58.0 ± 17.9^2^^,^^4^	74.7 ± 18.2	75.1 ± 17.5^4^
IL-6 in seminal plasma (ng/mL) ‡	114.6 ± 212.9	181.6 ± 295.7	100.7 ± 181.0	54.9 ± 53.4	57.5 ± 65.0
IL-8 in seminal plasma (ng/mL) §	19020.2 ±39499.8	25606.6 ±50821.9	8419.6 ±11826.2^1^	10913.9 ±16820.5	6966.5 ± 3031.3

^†^*TPV* total prostate volume, *Qmax* maximum urinary flow, *PSA* prostate-specific antigen, *I-PSS* International Prostate Symptom Score, *NIH-CPSI* NIH-Chronic Prostatitis Symptom Index, *QoL* quality of life, *8-EPI* 8-isoprostane, *IL-6* interleukin-6, *IL-8* interleukin 8

^‡^ sample sizes, Total: n = 34; Study group baseline: n = 16; Study group 4 weeks: n = 15; Control group baseline and 4 weeks: n = 18

^§^ sample sizes, Total: n = 29; Study group baseline: n = 16; Study group 4 weeks: n = 13; Control group baseline and 4 weeks: n = 13

^1^ *p*<0.05, within-group difference between baseline and week 4, Wilcoxon signed rank test

^2^ *p*<0.001, within-group difference between baseline and week 4, Wilcoxon signed rank test

^3^ p<0.05, between-group difference of change (week 4 –baseline), Mann-Whitney rank sum test

^4^ p<0.001, between-group difference of change (week 4 –baseline), Mann-Whitney rank sum test

### Effects on prostate-related parameters

In the study group, there was statistically significant improvement in Q_max_ and decrease of PSA levels in serum and NIH-CPSI and I-PSS total and sub-scores during the study period. In the control group, we found statistically significant changes in I-PSS sub-scores and NIH-CPSI total score (Table 1). The median values that changed the most were: I-PSS total score from 13(8–16) to 7.5(5–10) in the study group versus from 10(8–14) to 8(4–12) in the control group. The NIH-CPSI pain subscore changed from 6(2–7) to 1(0–4) in the study group versus from 5(2–8) to 3(0–5) in the control group.

When comparing the study group and the control group we found statistically significantly different changes of prostate-related characteristics, including PSA level in serum (p = 0.048), nocturnal and obstructive sub-scores (p = 0.02 for both), and a clear tendency for I-PSS total score (p = 0.051). The decrease of these parameters was more prominent in the study group, suggesting a favorable effect of FWP.

### Effects on systemic oxidative stress and inflammatory markers

In the study group, there was a statistically significant decrease of 8-EPI (p < 0.001) in urine and substantial decrease of IL-8 (p = 0.052) in seminal plasma. There were no statistically significant changes in oxidative stress and inflammatory markers in the control group.

The change of 8-EPI values was statistically significant between study and control group (p < 0.001). As median values may describe the changes better in some aspects we also did calculate the medians. The most prominent changes in medians were the following ones (from baseline to the endpoint 4 weeks later): 8-EPI in urine ng/mmol creatinine changed from 68.55(54-3-81.8) to 63.35(49.6–73.1) in the study group and from 76(59.8–88.5) to 73.8(60–90) in the control group. The amount of IL-6 (ng/mL) in seminal plasma changed from 65(34.4–192.8) to 37.1(8.9–102.7) in the study group and from 42.65(18.9–62.5) to 32.95(17.4–73.7) in the control group. The content of IL-8 in seminal plasma (ng/mL) changed from 9969.1(4959–21729.25) to 5231.7(1321.1–8399) in the study group and from 5177.4(2546.5–10077.7) to 3182.4(2245.3–7620.5) in the control group.

### Correlation and regression analyses to detect relationships between LUTS, systemic oxidative stress, age and prostate-related parameters

There was a statistically significant association between the change in pain score and the change of 8-EPI values in the study group (p = 0.004). This quadratic relationship between changes in pain scores and changes of 8-EPI values followed the curve with the coefficient of determination R^2^ = 0.446 (Fig 2).

The results showed a statistically significant correlation between the initial pain score and the change in pain score in the study group (r = -0.648; p = 0.001). The coefficient of determination was 0.467 and significance p < 0.001 for respective linear regression line (Fig 3). In addition, statistically significant correlations were found for changes of PSA with Q_max_ (r = -0.472; p = 0.027) and for changes of NIH-CPSI total score with 8-EPI (r = 0.432; p = 0.045) in the study group.

A multiple regression analysis was subsequently performed to uncover the effects of age and main prostate related parameters on LUTS. Multiple regression analysis based on the change between baseline and final values showed a significant effect for age to I-PSS obstructive sub-score (beta = -0.096, p = 0.021) and for 8-EPI to NIH-CPSI total score (beta = 0.238, p = 0.008) and NIH-CPSI pain sub-score (beta = 0.129, p = 0.027) in the study group. In addition, the multiple regression results for the baseline values of all enrolled subjects are presented in Table 2.

**Table 2:** Multiple regression analysis of LUTS^†^ (regression coefficients beta^‡^) at the baseline.

Characteristics^§^ (n = 43)	I-PSS total	I-PSS irritative	I-PSS obstructive	NIH-CPSI total	NIH-CPSI pain	NIH-CPSI QoL
Age (years)	0.0612	-0.0267	0.0611	-0.0267	-0.0120	0.0071
PSA (ng/mL)	-0.2212	-0.0508	-0.2032	-0.3450	-0.1589	-0.1432*
8-EPI (ng/mmol creatinine)	-0.0128	0.0041	-0.0183	0.0850	0.0405	0.0165
Qmax (mL/s)	-0.2641	-0.0785	-0.1845*	-0.1141	0.0295	-0.0188
TPV (mL)	0.0006	0.0220	-0.0079	0.0077	-0.0174	0.0121

^†^ NIH-CPSI and I-PSS scores were regressed on main prostate related parameters and age

^‡^ The beta coefficient indicates the association of the characteristic with corresponding scores

^§^ *PSA* prostate-specific antigen, *8-EPI* 8-isoprostanes in urine, *Qmax* maximum urinary flow, *TPV* total prostate volume, *I-PSS* International Prostate Symptom Score, *NIH-CPSI* NIH-Chronic Prostatitis Symptom Index, *QoL* quality of life

* *p* < 0.05 is considered as statistically significant

A multiple regression analysis on LUTS with age and main prostate related parameters based on the changes, including both groups, was performed to study interaction effect between the study and the control group. Significant difference between groups was detected in effect for 8-EPI to NIH-CPSI total score (beta = 0.238 and beta = -0.181 for study and control group respectively with difference of 0.419, p = 0.0289). Therefore, larger decrease of 8-EPI occurred with larger decrease of NIH-CPSI total score in study group, but with larger increase of NIH-CPSI total score in control group.

## Discussion

BPH is often associated with lower urinary tract irritative, obstructive and nocturnal symptoms. BPH is characterized by hyperplasia of prostatic stromal and epithelial cells, resulting in the formation of nodules in the periurethral region of the prostate. As BPH is often associated with prostatitis, the association between seminal inflammatory markers and prostate-related parameters in males with moderate LUTS was not surprising. These findings were comparable with our recent studies in middle-aged subjects.[12]

As a large number of men have moderate LUTS which impairs their general QoL the main goal of our study was to evaluate the effects of a FWP on LUTS and BPH parameters in men with moderate LUTS with special emphasis on NIH-CPSI pain sub-score. In addition, the effect of a special FWP on systemic OxS and seminal plasma inflammatory status was also investigated.

After consumption of FWP for 4 weeks Q_max_, PSA level, NIH-CPSI pain sub-score and a systemic OxS parameter (8-EPI) had statistically significant improvement and a substantial decrease of seminal plasma inflammatory marker (IL-8) occurred only in the study group. In the control group we had statistically significant changes in some scores in I-PSS and NIH-CPSI total scores (Table 2). The only significant changes in the control group were observed by questionnaire evaluation which is a subjective measurement and thus more likely to be influenced by a possible placebo effect. Changes in the questionnaire results in the study group were more prominent (statistically significant) than in the control group. Next, the only statistically significant differences between groups occurred for changes of 8-EPI, PSA and I-PSS obstructive sub-score. That finding may describe that male subjects with mild or moderate LUTS do not always need a pharmacological treatment and in that clinical situation it is important to understand the reason(s) of urinary dysfunction by a physician.

An improvement of the abovementioned indices can be explained by the hypothesis that FWPs have some antioxidant and/or anti-inflammatory properties due to whey proteins which are rich in BCAAs and cysteine, a precursor of glutathione synthesis. In addition, hydrogen sulfide synthesized from cysteine may cause bladder relaxation and thus be associated with LUTS[15].

FWP mechanism of action could possibly be mediated by an elevation of GSH concentration and a decreased glutathione redox ratio (GSSG/GSH) ratio which leads to a suppression of OxS and the decreased release of 8-EPI. Hydrolyzed whey protein isolate can increase intracellular glutathione level in human prostate epithelial cell line and protect form oxidant induced cell death[16].

The additional mechanism could be the growth promotion of the beneficial bacteria as shown in a study with a whey-based infant formula.[17] As our FWP also contains a certain amount of several peptides derived from whey proteins that may decrease infiltration of leucocytes and thus reduce the production of reactive oxygen species (ROS).

Suppression of OxS by hydrolyzed whey protein may have a role in the improvement of QoL of men with LUTS because it is possible that there may be a connection between systemic OxS (8-EPI) and pain receptors. One of the most interesting findings was the statistically significant quadratic relationship between the decrease in the pain score and 8-EPI in urine after FWP consumption (Fig 2). The non-linear nature of this relationship is caused by the circumstance that in the case of small changes in pain, 8-EPI changes have higher variability around the prognostic curve. It can be explained by the fact that the pain scale has no continuous measurement. Next, there was the statistically significant linear relationship between the initial value of the pain and decrease of the pain score in the study group (Fig 3). This finding shows that the most severe pain at the beginning of the trial resulted in the bigger decrease of pain score.

Prostaglandin-like compounds are synthesized during OxS-related processes and seem to induce bladder over-activity as shown in rabbits. One of these products, isoprostanes-8-epi PGF2α, is released during bladder contraction and induces a dose-dependent bladder smooth muscle contraction. Isoprostanes are electrophilic lipid peroxidation products (LPP) that form by non-enzymatic reaction between arachidonic acid and ROS. Measurement of 8-EPI (the products of lipid peroxidation) in urine is a reliable approach to assess systemic oxidative stress *in vivo* because of their relative stability. Isoprostanes also contribute to pain reaction because they can up-regulate nociceptive pathways by stimulating prostanoid receptors. A detailed mechanism of a hypothetical LPP-pain circle has been published before. For illustrative purposes see Supporting Information Fig 1 that has some parts previously published in [18]). Briefly, prostate, bladder and urethra innervation pathways express nociceptors TRPA1 (transient receptor potential cation channel, subfamily A, member 1) and TRPV1 (transient receptor potential cation channel, subfamily V, member 1).[19–20] Electrophilic LPP are relevant because they activate nociceptor TRPA1 and react with intracellular thiols including glutathione, a crucial nonenzymatic intracellular antioxidant.[21–22] Therefore, peripheral stimuli (initial pain) can lead to spinal OxS, which is the key link in pain-causing pathways [23].

Three positive feedback pathways that stem from spinal OxS (lipid peroxidation due to uncompensated superoxide overproduction) are elaborated below and discussed in detail in [18, 23]. The first pathway consists of GSH depletion by electrophilic LPP. An animal experiment has shown that the pain induced in such a way can be reversed by administration of GSH.[24] The second pathway might be mediated by electrophilic LPP, which can bind with TPRA1 in primary sensory afferents. Therefore, the OxS in the dorsal horn of the spinal cord provides positive feedback for painful stimuli.[25–26] The third pathway is mediated by LPP that also can be ligands of prostanoid receptors. These ligands include 8-EPI and can be sensitized by peripheral 8-EPI and spinal PGF2α (S1 Fig). [27–28]

The present study has a few limitations. While the main goal of our study was to investigate the effects of FWP in patients with moderate LUTS, a challenging problem was to compose an optimal protocol for the study to assess all parameters related to urinary function in men >30 years. We tried to minimize the possible weaknesses, including the screening of semen. For example, almost three-quarters of the previous studies of seminal parameters in middle-aged men did not state the duration of abstinence before semen analysis.[29] In our study, the recommended period of abstinence was no shorter than 48 hours and no longer than seven days for all participants. In the initial phase of the study, the subjects with a reported incomplete semen sample were excluded.[8]

Also we only did have two time points so we can not say anything about the dynamics or persistence of the effect.

Finally, the present group included only men who attended a screening in the outpatient clinic and agreed to provide semen specimens, and therefore they do not represent directly the general population of men aged >30 years.[12, 30]

## Conclusion

The consumption of a special FWP improved urinary function and reduced LUTS. Whereas especially remarkable were the decrease of the PSA level in serum and the decrease of 8-EPI in urine concomitant with pain reduction. In summary, a special FWP may contribute to an improvement of the quality of life in males with moderate LUTS. However, a longer treatment period should maybe be applied in the further studies.

## Supporting information

S1 File The materials (translated to English) presented to the ethics committee—Provided as an answer to a reviewer. (DOC)

S2 File The CONSORT type checklist of the submitted article. (DOC)

S1 Table The content of the whey-based product and the control drink. (DOC)

S2 Table The complete table of individual subjects´ results. (XLSX)

S1 Fig Putative vicious circles mediated by lipid peroxidation products, including isoprostanes. Putative vicious circles mediated by lipid peroxidation products, including isoprostanes. (Originally published by Türk and Kullisaar in *Med Hypotheses*. 2011; **77**: 837–40)
Prostate pain is sufficient to cause neural OxS.OxS in the dorsal horn of the spinal cord provides a positive feedback for painful stimuli, as dorsal horn neurons secrete bioactive LPP. Whereas 8-isoprostanes and PGF2a sensitize the primary sensory afferents, electrophilic LPP can excite them directly via TRPA1.Neural OxS contributes to systemic OxS by competing for glutathione precursors (cystine and cysteine) and by exporting LPP (8-isoprostanes) that are released into the blood and eventually into the urine.Contractile bioactivity of 8-isoprostanes in urine may determine whether other urinary tract pathologies remain subclinical or become symptomatic.
(DOCX)

## Abbreviations

8-EPI: 8-isoprostanes
BCAAs: branched-chain amino acids
BPH: benign prostatic hyperplasia
FWP: fermented whey product
GSSG/GSH: glutathione redox ratio
IL-6: interleukin-6
IL-8: interleukin-8
I-PSS: International Prostate Symptom Score
LPP: lipid peroxidation products
LUTS: lower urinary tract symptoms
NIH-CPSI: National Institutes of Health Chronic Prostatitis Symptom Index
OxS: oxidative stress
PSA: prostate-specific antigen
Q_max_: maximum urinary flow
QoL: quality of life
ROS: reactive oxygen species
TPV: total prostate volume
