# Supplementary material for: Fermented whey-based product improves the quality of life of males with moderate lower urinary tract symptoms: A randomized double-blind study
Source: PLoS One. 2018 Feb 23;13(2):e0191640. doi: 10.1371/journal.pone.0191640 (PMC5825006; doi:10.1371/journal.pone.0191640)
Supplement: S1 File — (DOC) [file pone.0191640.s001.doc]

1. The name of the study:

An investigation of the improved whey-based fermented produkt in males with moderate urinary dysfunction (IPSS score<19).

The study is an improved extension of another project (ethics committe approvement 22.02.2010, protocol nr. 190T-1)

The components of the planned investigation.

1. The base of the study is whey that was fermented with lactobacilli *(L. plantarum* MCC1, that is a Basic jogurt-maker, and *L. gaseeri* MCC2 that is isolated from a helthy human GI-tract); arter fermentation the lactobacilli were killed by pasteurization – the produkt did not contain live bacteria.
2. Pumpkin oil – Manufacturer B.Schell GmbH, Speiseöle. D-77839 Lichtenau/Baden Germany. Contence: per 100g product energy 900 kcal, lipids 100 g. Does not contain arome- or flavour additives, preservatiives. Manufactured from pumpki seeds with cold-pressing methods. 100% natural product.
3. Rye bran – Manufacturer Vändra Leib LLC. Per 100 g: energy 605 kJ / 147 kCal, proteins 13.0 g, carbohydrates 15.0 g, lipids 2.6 g. Originates from ecological agriculture.
4. Apricot-mango additive – Manufacurer Saarek LLC. Per 100 g product: energy 976 kJ / 233 kcal, proteins 0.1 g, carbohydrates 59.6 g, lipids 0 g. Contence: sucrose, water, glucose-fructose syrup, apricots (10 %), mangos (10 %), acidity regulator: E330, paksendaja: E440, natural flavoring agents, color: E160a.

**References :**

1. Noraini M. Knhalid and Elmer H. Marth. 1990 Proteolytic activity by strains of Lactobacillus plantarum and Lactobacillus casei. J Dairy Sci. 73, 3068-3076
2. Bottazzi, V. Other fermented dairy products. Food and feed production with microorganisms. In: Biotechnology. (G. Reed, edit.) Verlag Chemie, Weinhein, 5, (1983), pp. 315−364.
3. Mikelsaar, M., Annuk, H., Shchepetova, J., Mändar, R., Sepp, E. and Björksten, B. (2002). Intestinal Lactobacilli of Estonian and Swedish children. Microb. Ecol. Health Dis. 14:75-80.
4. Kullisaar, T, Songisepp, Mikelsaar M, Zilmer, K, Vihalemm, T, Zilmer, M. Anti-oxidant probiotic fermented milk decreases oxidative stress-mediated atherogenicity in human. *British J of Nutrition*. 2003, 90,2, 449-456.
5. Songisepp E, Kullisaar T, Hütt P, Elias P, Brilene T, Zilmer M, Mikelsaar M. A New Probiotic Cheese with Antioxidative and Antimicrobal Activity. J. Dairy Sci. 2004, 87, 2017-2023.
6. *Feedinfo- Scientific Reviews* – Online. A new probiotic cheese with antioxidative and antimicrobial activity. Mikelsaar, M, Zilmer, M. 2004, 44, 1-4.
7. Songisepp, E., Kals, J., Kullisaar, T., Hütt, P., Mändar, R., Zilmer, M., Mikelsaar, M. Evaluation of the functional efficacy of a probiotic in healthy volunteers. Nutrition Journal, 2005, 4:22.
8. *Feedinfo- Scientific Reviews* – Online. A new probiotic cheese with antioxidative and antimicrobial activity. Mikelsaar, M, Zilmer, M.2004, 44,1-4.
9. Third Central European Congress on Food, May, 2006, Sofia, Bulgaria. Consumption of antioxidative probiotic probiotic *L. fermentum ME-3* and its impact on post-prandial lipid profile in human volunteers. Kullisaar, T, Zilmer, K, Vihalemm, T, Zilmer, M. pp. 208.
10. T. Kullisaar, K. Zilmer, M. Mikelsaar, E. Songisepp and M. Zilmer Probiotics may have beneficial effects on both the postprandial oxidative stress and lipid profile status in humans. Br J Nutrition 2003, 90, 449-456.
11. Songisepp, E., Rätsep, M., Üksti J., Zilmer K., Hütt, P., Mikelsaar M. „The improvement of cheese quality with human *Lactobacillus plantarum* additive” 1^st^ open seminary arranged by SAFOODNET “Microbial Contaminants and contamination routes in food industry” at VTT in Espoo, Finland, January 22-23, 2007.
12. T. Kullisaar, S. Türk, M. Punab, P. Korrovits, K. Kisand, A. Rehema, K. Zilmer, M. Zilmer & R. Mändar Oxidative stress in leucocytopermic prostatitis patients: preliminary results. Andrologia 2007, 39, 1-12.
13. EFSA Panel on Dietetic Products, Nutrition and Allergies (NDA) Scientific Opinion on the substantiation of health claims related to polyphenols in olive and protection of LDL particles from oxidative damage. EFSA Journal 2011;9 (4):2033[25 pp.]. doi:10.2903/j.efsa.2011.2033; [www.efsa.europa.eu/efsajournal](http://www.efsa.europa.eu/efsajournal)

Estonian patent application EE201100012 "Isolated strains of microorganisms MCC1 DSM23881 and MCC2 DSM23882 and the use thereof" (unpublished), applicant Bio-Competence Centre of Healthy Dairy Products, inventors Tiiu Kullisaar, Mihkel Zilmer, Ene Tammsaar, Andre Veskioja, Epp Songisepp, Kersti Zilmer, Lauri Bobrovski, Kersti Ehrlich, Merle Rätsep, Margus Punab, Maire Vasar, Marika Mikelsaar.

**8. Planned time frame of the investigation**

May 2012– December 2014

**9. Recruition and selection of the subjects**

The selection of subjects takes place in Medita clinic among out-patients. We plan to recruite 72 males, 45-75 years of age.

The investigation group includes males with moderate dysuria (IPSS 8-19) by International Prostate Symptome Score.

We include men with the following clinical prameters:

PSA value <10 ng/ml;

- Speed of urinary flow 5-15 ml/s;
- Prostate volume <80 mL
- Residual urine measured by abdominal ultrasound <300 mL

We exclude men with the following clinical parameters:
- tumorous changes in urogenital tract;

- acute colic type pain originating from urogenital tract;

- pelvic region radiation or chemotherapy in anamnesis;

- nodulous structural change in the prostate detected by palpation that can hint on a tumor

- prostatitis (NIH II, NIH IIIA, NIH IV, but not NIH IIIB)

We also exclude men who:

- have had prostate surgery;

- have used α_1_- adrenoblocking medication in 2 weeks;

- has used 5α-reductase inhibitors in recent 5 months.

**10. The informed consent forms**

**11. Description of methodology**

The matter of investigation is a fermented whey-based milk product that is specially manufactured ( small-scale) in the frame of a scientific joint project of Tartu University Institute of Biochemistry and Bio-Competence Centre of Healthy Dairy Products LLC . The clinical investigation is planned to be double-blind and randomized.

Both the whey-based fermented product and control liquid are packaged in similar non-transparent coded batches. The codes are only known to the manufactorer and he is not a part of the following procedural steps of the investigation. All participants (subjects) of the investigation are given a numerical code and the analyses later performed on the patient-derived materials are not later connected to individual persons data. All passwords are allocated to standard password-protecting rules. Only responsible processor of data can sign contracts/ agreements with authorized processors of data with all the needed agreements of confidentiality and only in the limitations of strict needs of the investigation responsibilities. The data that can lead to individual personal data of the subjects are destroyed by the responsible processor of data after the results are published.

The intervention group will consume 200 g of whey-based fermented product for 4 weeks, the control group will consume 200g of apple juice for 4 weeks. All participants will provide serum samples, urine samples and seminal plasma before and after the 4-week test period. On both occasions 2 questionnares (IPSS and general symptoms questionnaire) are also fulfilled by the participants.

The investigation contains the following blocks:

1. Medical examination and biochemical-clinical parameters from blood plasma, urine and seminal plasma: clinical blood, triglycerides, cholesterol fractions, usCRV, PSA ALT, AST, glucosylated hemoglobin, vitamin D, urine creatinine, leptin, testosterone, androgene binding protein. Also ultrasound, palpation, BMI, fat percentage measurement will be performed.

2.Specific biochemical-metabolomic part of specific markers in blood and urine: interleucines, growth factors, oxidative stress index (OSI), oxLDL, 8-isoprostanes, glutathione redox-ratio, myeloperoxidase MPO, adiponectin.

3. Markers of metabolic syndrome: blood pressure; fat content of the body; adiponectii, leptin, insulin

4. Metabolomics markers: 4…6 target-metabolites.

5. Comet assay that enables to quantify the DNA damage in sperms.

6. Genetic investigation.

For the analysis of DNA the participants give (2 9-ml EDTA-test tubes) venous blood once. The blood is driven by an experienced nurse. The test tubes are coded and dated and sent to the lab for further investigation. DNA will be extracted from Ahole blood by standard precipitation with saline buffer. DNA will be stored in TE (Tris-EDTA) solution in Tartu University Institute of Physiology/biochemistru at -20⁰C. For genotyping the DNA polymorphisms several methods can be used (e.g. tetra-primer ARMS-PCR protocol, where DNA is amplified with PCR and the presence of products is checked by gel). Allel- and haplotype determinations will be performed by Haploview programme.

**The informed consent:**

Name of the investigation: An investigation of the improved whey-based fermented produkt in males with moderate urinary dysfunction (IPSS score<19).

We ask You to join an joont investigation conducted by Tartu University Institute of Biochemistry, Bio-Competence Centre of Healthy Dairy Products LLC and MediTa.

Please read the following text carefully and ask all your questions from our investigators andrologist dr. K. Ausmees (phone:. 56 458 430, e-mail: [**kristo.ausmees@medita.ee**](mailto:kristo.ausmees@medita.ee) and dr. G. Timberg (phone: 56 458 430, e-mail: [**gennadi.timberg@medita.ee**](mailto:gennadi.timberg@medita.ee))

Wat is the purpose of the study?

The purpose of the investigation ist o explore the effect of whey-based fermented product on males with moderate urinary tract complaints, who have mainly irritative symptoms and do not have an inflammation .

What is tested and how?

A group of patients (investigation group) is going to use 200g of a whey-based product for 4 weeks, the subjects in the control group are going to use apple juice 200 g/day. Before the test period the subjects give blood, urine and sperm plasma for analysis, the tests are repeated after the test period in 4 weeks. 2 questionnaires have to be fulfilled three times as well (General questions questionnaire, supportive material No2 and IPSS (International Prostate Symptom Scale), Supportive Material No1)

The investigation contains the following blocks:

1.Clinival investigations and the corresponding biochemical-clinical blood plasma, urine and seminal plasma markers: clinical haemogramme, triglycerides, cholesterol fractions, usCRV, PSA, ALT, AST, glucosylated hemoglobin, vitamin D, urine kreatinin, leptin, testosterone, usCRP. You will also be subjected to the following clinical investigations: ultrasound, palpation, measurement of BMI, measurement of body composition (fat of total body weight).

2.Specific biochemical-metabolomic part of specific markers in blood and urine: interleucines, growth factors, oxidative stress index (OSI), oxLDL, 8-isoprostanes, glutathione redox-ratio, myeloperoxidase MPO, adiponectin.

3. Markers of metabolic syndrome: blood pressure; fat content of the body; adiponectii, leptin, insulin

4. Metabolomics markers: 4…6 target-metabolites.

5. Comet assay that enables to quantify the DNA damage in sperms.

6. Genetic investigation.

For the analysis of DNA the participants give (2 9-ml EDTA-test tubes) venous blood once. The blood is driven by an experienced nurse. The test tubes are coded and dated and sent to the lab for further investigation. DNA will be extracted from Ahole blood by standard precipitation with saline buffer. DNA will be stored in TE (Tris-EDTA) solution in Tartu University Institute of Physiology/biochemistru at -20⁰C. For genotyping the DNA polymorphisms several methods can be used (e.g. tetra-primer ARMS-PCR protocol, where DNA is amplified with PCR and the presence of products is checked by gel). Allel- and haplotype determinations will be performed by Haploview programme.

During the first visit You will be given the information about the investigation and the time of taking analyses will be agreed. At the beginning and in the end of the investigation You will be giving the blood Sampole, urine and sperm sample (seminal plasma will be extracted later from the provided material). Your PSA value, urinary flow speed, prostate volume and residual urine volume (abdominally measured) will be measured.

During the investigation You are goung to fulfil the questionnaires three times: one week before the „zero-point“, in 1 week and in the last week.

Men with these conditions are not included into the study:
- tumorous changes in urogenital tract;

- acute colic type pain originating from urogenital tract;

- pelvic region radiation or chemotherapy in anamnesis;

- nodulous structural change in the prostate detected by palpation that can hint on a tumor

- have had prostate surgery;

- have used α_1_- adrenoblocking medication in 2 weeks;

- has used 5α-reductase inhibitors in recent 5 months.

When coming to the clinic for investigation meeting You should not eat, use any medication, alcohol, coffee or tea nor smoke for 12 hours. If needed, additional information will be provided by dr. K. Ausmees (phone: 56 458 430, e-mail: [kristo.ausmees@medita.ee](mailto:kristo.ausmees@medita.ee) or dr. G. Timberg (phone: 56 458 430, e-mail: [gennadi.timberg@medita.ee](mailto:gennadi.timberg@medita.ee)).

In the morning of the investigation meeting day in the MediTa clinic an experienced nurse will draw 20 ml venous blood from You, You´ll also provide an urine and sperm sample (by masturbation), from which different biomarkers will be measured.

After this You will be given 28 daily doses of whey-based product to consume daily. After 4 weeks the investigative procuders will be repeated.

Can this investigation be dangerous in any way?

We can not exclude the possibility that after taking the blood Sampole ther develops a local hematoma and tenderness in the location of venopuncture. In general, taking blood saples by an experienced MediTa nurse is safe, also providing sperm analysis and the ultrasound investigation are harmless, although we can not exclude minimal senise of unpleasantness.

If needed, additional information will be provided by dr. K. Ausmees (phone: 56 458 430, e-mail: [**kristo.ausmees@medita.ee**](mailto:kristo.ausmees@medita.ee) or dr. G. Timberg (phone: 56 458 430, e-mail: [**gennadi.timberg@medita.ee**](mailto:gennadi.timberg@medita.ee)).

What information will this investigation provide?

The subjects of the investigation are allocated to more extensive and thorough than routine clinical analyses of general and prostate healt. This innovative multi-disciplinary project integrates different investigations. The results of this investigation may lead to a possibility to improve the diagnostic and treatment methods, empower the prophylactic measures concerning male reproductive health and thus improve the quality of life of males, maybe even increase male life expectancy and diminish social and Medial expenditure.

The investigation project is safe and may ease Your healt isuses. Also by participating You will be helping in a scientific project that has a goal of developing new innovative healt-improving foods.

If I want to withdraw my participation?

*You are in no way obliged to take part in the investigation. Your participation is totally voluntary. You may discotinue the participation in the investigation at any moment without any negative consequences. Also the doctor has te right to eliminate You from the study at any moment if it turnes out You do not suit into the investigation group, do not follow the investigation protocol or it is needed for Your best interests.*

Confidentiality

All Your data will be handled confidentially and will be used only for scientific purposes. In documents, data processing and publications only your study code, not a name will be used.

Who to contact when I have questions?

If needed, additional information will be provided by dr. K. Ausmees (phone: 56 458 430, e-mail: [**kristo.ausmees@medita.ee**](mailto:kristo.ausmees@medita.ee) or dr. G. Timberg (phone: 56 458 430, e-mail: [**gennadi.timberg@medita.ee**](mailto:gennadi.timberg@medita.ee)).

AN INFORMED CONSENT FORM

An investigation of the improved whey-based fermented produkt in males with moderate urinary dysfunction (IPSS score<19).

I have read through the patient information list and understood the contence.

I have asked questions if needed and consulted the details of the investigation with the coordinator of the investigation. After completing the investigation I will get the information about my health matters from my andrologist (dr. K Ausmees or dt. G. Timberg).

Taking the venous blood samples takes place during an agreed out-patient doctor´s apointment by an experienced MediTa clinic nurse. During the same visit a sperm sample will also be provided (the sperm plasma will be extracted from the masturbation-produced sample), also an ultrasound investigation, urinary flow measurement and measurement of prostate volume.

All men participating in the investigation are guaranteed to get total confidentiality concerning their personal data. All participants will be given a numeric code and and the analyses later performed on the patient-derived materials are not later connected to individual persons data.

When processing and storing the personal data concerning this project secured (virus- and spywere-protected) digital data carriers are used. For entering the system all users have to identify them by personal user names and passwords/ ID_cards. For passwords strong password-protecting rules apply. All passwords are allocated to standard password-protecting rules. Only responsible processor of data can sign contracts/ agreements with authorized processors of data with all the needed agreements of confidentiality and only in the limitations of strict needs of the investigation responsibilities. The data that can lead to individual personal data of the subjects are destroyed by the responsible processor of data after the results are published. All subjects will be given feedback about the results of the clinical investigations made in connection to this investigation. Participation in the investigation is voluntary and I can withdraw from the project at any moment without explanation.

Based on the provided information I do agree in participating in the investigation.

By signing this informed consent form I have not given up any of my legal rights that I would have had without participaing in this investigation.

_____________________________ _______________________

Signature of the patient Date

_______________________________________________________

Patsient´s name in block letters

The Patient has been instructed about the possible hazards concerning the investigation and the nature of the conducted procedures nature and purpose. .

_____________________________ _______________________

Signature of the Doctor Date

______________________________________________________

Name of the doctor in block letters: dr G. TIMBERG (phone: 56 458 430, email: [gennadi.timberg@medita.ee](mailto:gennadi.timberg@medita.ee)) and dr. K. AUSMEES (phone: 56 458 430, email: [kristo.ausmees@medita.ee](mailto:kristo.ausmees@medita.ee)).

**QUESTIONNAIRE**

**GENERAL QUESTIONS – Additional file 2**

**Age:** …………… **Sex**:  Male  Female

**General feeling**

5* 4 3 2 1 0

*Please evaluate on scale (1 - 5), 5 = the best, 1= the worst, 0= non applicable

**APPETITE** Remarks:…………………………………………………………

Bad appetite  normal  elevated appetite

**NAUSEA** Remarks:…………………………………………………………

None  Sometimes  Conctant  Taking medication against nausea

**VOMITING**

Yes  No

**DIGESTION / Bowel activity**

Normal  Constipation  Diarrhea  Use of laxatives

**Normal bowel activity**– defecation 3 times a day till 3 times a week.

**Constipation** – less than 3 times a week.

**Diarrhea** – more than 3 times a day and feces is liquidish.

**Feeling full**  – dyscomfort feeling after a meal.

**Flatulence** – excess gases in the gastrointestinal trakt, may cause reeling full.

**Eating habits**

Vegan

Mixed food eater

| A week before the investigation | **1.week** |
| --- | --- |
| **General feeling**  5 very good  4 good  3 so-so  2 bad  1 very bad  0 not applicable | **General feeling**  5 very good  4 good  3 so-so  2 bad  1 very bad  0 not applicable |
| **Appetite**  better  no change  worsened | **Söögiisu**  better  no change  worsened |
| **Nausea**  none  slight  moderate (interferes everyday life)  strong (incapable for normal activities) | **Iiveldus**  none  slight  moderate (interferes everyday life)  strong (incapable for normal activities) |
| Vomiting No  Yes …..(times a day) | Vomiting No  Yes …..(times a day) |
| Defecation …………….**(times a day)** | Defecation …………….**(times a day)** |
| **Defecation**  easy (normal)  needs effort | **Defecation**  easy (normal)  needs effort |
| **Fecal consistency**  liquid  normal  hard  softer | **Fecal consistency**  liquid  normal  hard  softer |
| **Fecal volume**  a lot  a little  fibrous | **Fecal volume**  a lot  a little  fibrous |
| **Abdominal pain**  none  slight  moderate  strong | **Abdominal pain**  none  slight  moderate  strong |
| **Flatulence**  none  mild  moderate  strong | **Flatulence**  none  mild  moderate  strong |
| **Feeling full**  no  mild  moderate  strong | **Feeling full**  no  mild  moderate  strong |
| Other allergy  fever ...............  use of medication ……………… | Other allergy  fever ...............  use of medication ……………… |
| Notes ……………….. | Notes ……………….. |

| 4.week |  |
| --- | --- |
| **General feeling**  5 very good  4 good  3 so-so  2 bad  1 very bad  0 not applicable |  |
| **Appetite**  better  no change  worsened |  |
| **Nausea**  none  slight  moderate (interferes everyday life)  strong (incapable for normal activities) |  |
| Vomiting No  Yes …..(times a day) |  |
| Defecation …………….**(times a day)** |  |
| **Defecation**  easy (normal)  needs effort |  |
| **Fecal consistency**  liquid  normal  hard  softer |  |
| **Fecal volume**  a lot  a little  fibrous |  |
| **Abdominal pain**  none  slight  moderate  strong |  |
| **Flatulence**  none  mild  moderate  strong |  |
| **Feeling full**  no  mild  moderate  strong |  |
| Other allergy  fever ...............  use of medication ……………… |  |
| Notes ……………….. |  |
