# Supplementary material for: Fermented whey-based product improves the quality of life of males with moderate lower urinary tract symptoms: A randomized double-blind study
Source: PLoS One. 2018 Feb 23;13(2):e0191640. doi: 10.1371/journal.pone.0191640 (PMC5825006; doi:10.1371/journal.pone.0191640)
Supplement: S1 Table — (DOC) [file pone.0191640.s003.doc]

**Supportive information Table 1** Chemical parameters of whey and the end product (fermented whey product)

| **Ingredient/parameter** | **Whey**  Mean ± SD | **Product**  Mean ± SD |
| --- | --- | --- |
| Fat (%) | < 0.1 | 0.72 ±0.03 |
| Protein (%) | 0.66 ± 0.10 | 0.98 ± 0.04 |
| Carbohydrates (%) | 4.07 ± 0.59 | 9.9 ± 1.2 |
| Dry matter (%) | 5.37 ± 0.27 | 11.99 ± 0.12 |
| pH | 6.14 ± 0.41 | 3.99 ± 0.14 |
