# Supplementary material for: Fermented whey-based product improves the quality of life of males with moderate lower urinary tract symptoms: A randomized double-blind study
Source: PLoS One. 2018 Feb 23;13(2):e0191640. doi: 10.1371/journal.pone.0191640 (PMC5825006; doi:10.1371/journal.pone.0191640)
Supplement: S1 Fig — Putative vicious circles mediated by lipid peroxidation products, including isoprostanes. (Originally published by Türk and Kullisaar in Med Hypotheses. 2011; 77: 837–40) Prostate pain is sufficient to cause neural OxS.OxS in the dorsal horn of the spinal cord provides a positive feedback for painful stimuli, as dorsal horn neurons secrete bioactive LPP. Whereas 8-isoprostanes and PGF2a sensitize the primary sensory afferents, electrophilic LPP can excite them directly via TRPA1.Neural OxS contributes to systemic OxS by competing for glutathione precursors (cystine and cysteine) and by exporting LPP (8-isoprostanes) that are released into the blood and eventually into the urine.Contractile bioactivity of 8-isoprostanes in urine may determine whether other urinary tract pathologies remain subclinical or become symptomatic. (DOCX) [file pone.0191640.s005.docx]

**Supportive Information Figure 1** Putative vicious circles mediated by lipid peroxidation products, including isoprostanes. (Originally published by Türk and Kullisaar in *Med Hypotheses*. 2011; **77**: 837-40)

(1) Prostate pain is sufficient to cause neural OxS.

(2) OxS in the dorsal horn of the spinal cord provides a positive feedback for painful stimuli, as dorsal horn neurons secrete bioactive LPP. Whereas 8-isoprostanes and PGF2a sensitize the primary sensory afferents, electrophilic LPP can excite them directly via TRPA1.

(3) Neural OxS contributes to systemic OxS by competing for glutathione precursors (cystine and cysteine) and by exporting LPP (8-isoprostanes) that are released into the blood and eventually into the urine.

(4) Contractile bioactivity of 8-isoprostanes in urine may determine whether other urinary tract pathologies remain subclinical or become symptomatic.
